# Sampling Methodology to Maximize the Efficient Use of National Abattoir Surveillance: Using Archived Sera to Substantiate Freedom From Bluetongue Virus Infection in Ireland

**DOI:** 10.3389/fvets.2018.00261

**Published:** 2018-10-24

**Authors:** Jamie A. Tratalos, Damien J. Barrett, Tracy A. Clegg, Ronan G. O'Neill, Guy McGrath, Elizabeth A. Lane, Simon J. More

**Affiliations:** ^1^Center for Veterinary Epidemiology and Risk Analysis, University College Dublin, Dublin, Ireland; ^2^Department of Agriculture, Food and the Marine, Dublin, Ireland

**Keywords:** bluetongue, surveillance, freedom from disease, serology, Ireland, sampling

## Abstract

In recent years, there has been increasing recognition of the value of multiple data sources available to fulfill surveillance objectives, and the use of these has been applied to address many questions relating to animal health surveillance. In Ireland, we face a slightly different problem, namely, best use of an existing surveillance resource (serological samples collected over many years from cull cows at slaughter), which has been used to substantiate freedom from *Brucella abortus* following its successful eradication in 2009. In this study, we evaluate a sampling methodology to use this resource to substantiate freedom from bluetongue virus (BTV) infection. An examination of the degree to which cull cows were resident in the same herd throughout the midge biting season showed that, of 50,640 samples collected between 17 October and 23 December 2016, 80.2% were from animals resident in the same herd between 01 April 2016 and 2 months prior to their slaughter date, 74.1% for 1 month prior, 70.1% for 2 weeks prior, 66.4% for 1 week prior, and 56.4% up to 1 day prior to slaughter. An examination was made of the degree to which individual samples within the same 88-well frozen storage block came from geographically clustered herds, whether from a concentration of animals from the same herd in a single block, or from clustering around the slaughterhouse where the samples were taken. On the basis of these analyses, a sampling strategy was derived aimed at minimizing the number of storage blocks which needed to be thawed, whilst ensuring a large enough and representative sample, geographically stratified according to the bovine population of 51 squares, each 45 × 45 km, covering the entirety of Ireland. None of the 503 samples tested were positive for BTV, providing reassurance of national BTV freedom. More broadly, the study demonstrates the use of abattoir-based serological samples collected for one large scale surveillance programme in surveillance for other bovine infections.

## Introduction

In 2006, several Northern European countries, including Belgium, Germany and the Netherlands, as well as France, experienced outbreaks of bluetongue caused by bluetongue virus serotype 8 (BTV8). The bluetongue virus (BTV) spread throughout Europe in the following year, including the reporting of cases in the UK, which marked a considerable expansion northwards from BTV's usual geographic range (1–4). Although vaccination and movement restrictions eliminated or controlled many of these outbreaks, BTV8 became established in parts of France in 2015, and BTV serotype 4 (BTV4) also emerged there in 2017 (5, 6). Both of these outbreaks are currently (June 2018) ongoing. The virus is mainly spread by biting *Culicoides* spp. “midges” (7, 8), which in Northern Europe generally occur during the summer months, when midges are most active. The virus can arise in new locations through wind dispersal of midges (9, 10), mediated by movements of an infected ruminant or camelid host or, theoretically, through importation of plants hosting vector midges (11). Although BTV infection in temperate climates is distinctly seasonal, it is thought that the virus may be able to overwinter in a new location once it has become established, although the precise mechanisms of this are not fully understood (7, 12).

Following introduction, BTV can spread quickly and cause substantial economic losses due to mortality and reduced production amongst cattle and sheep, as well as considerable surveillance, control, and vaccination costs (11, 13, 14). Within the European Union, confirmation of a case of bluetongue leads to the restriction of ruminant movements within a zone 150 km from the location of the case (EU Directive 2000/75/EC).

BTV8 did not reach the Republic of Ireland (henceforth “Ireland”) in 2006 and it has remained BTV free over the intervening period. However, given Ireland's proximity to, and frequent trade with, northern France, and also due to the possibility of outbreaks spreading from France to the UK, which is much closer and a more frequent trading partner, Ireland needs to be vigilant to the possibility of the incursion of BTV, whether through importing ruminant livestock or active or windborne dispersal of infective midges. Furthermore, Ireland needs to confirm freedom from BTV to enable the live export of ruminants, and products derived from them.

EU Commission Implementing Regulation (EU No 456/2012) Annex I, 3 stipulates criteria under which member states can demonstrate freedom from BTV infection using serological or virological surveillance. This needs to consist of random or targeted testing, performed at the time of year when seroconversion is likely to be detected, i.e., following the annual vector competent season. Sampling must be conducted such that samples are representative of the bovine population in the member state to be tested. The sample size must be sufficient, at a minimum, to detect a prevalence of 5% with a 95% confidence level. Furthermore, sampling should be stratified according to the spatial distribution of the bovine population, at 45 × 45 km resolution. In previous years, testing for BTV was conducted under a field-based sampling method conducted alongside Ireland's brucellosis testing programme. With that programme substantially reduced now that brucellosis has been eradicated, there was a need to find an alternative method to prove freedom from BTV infection.

In recent years, there has been increasing recognition of the value of multiple data sources available to fulfill surveillance objectives (15, 16), and these methods have been applied to address many questions relating to animal health surveillance [for example, (17, 18)]. Furthermore, many studies have evaluated the relative costs and effectiveness of different surveillance options. Welby et al. (2017) evaluated the effectiveness and cost-efficiency of different surveillance methods for demonstrating freedom from, and early detection of, BTV8 in Belgium, France and the Netherlands, and recommended an adaptive approach to surveillance depending on the stage of the epidemic (19). In Switzerland, Schärrer et al. (20) showed that slaughterhouse monitoring could be an equally or more cost-efficient method for monitoring animal-level disease prevalence or proving freedom from disease when compared to random on-farm sampling (20).

In Ireland, we face a slightly different problem, namely, efficient use of an existing surveillance resource: serology collected over many years from cull cows at slaughter. In addition to its cost effectiveness, this resource facilitates access to a large number of animals from a wide range of holdings throughout the country. For many years, sera from > 50,000 cows, from both dairy and beef herds, have been tested annually for case-finding for brucellosis (21) and, following the successful eradication of brucellosis from Ireland in 2009 and from the whole island of Ireland in 2015, to substantiate freedom from *Brucella abortus*. The cull cow monitoring programme was extended to most slaughterhouses in 2000 but has evolved with changing surveillance priorities. In combination with the cull cow data, the presence of a robust registration and movement database in Ireland facilitates the traceability of animals to the herds from which they were slaughtered and to all herds in which they resided over the course of their lifetime.

There will be an ongoing need to continue to substantiate freedom from brucellosis, but also BTV8 and enzootic bovine leukosis. Furthermore, case-finding for bovine viral diarrhea will be increasingly important, with ongoing progress toward national eradication (22), and potentially also with Johne's disease and infectious bovine rhinotracheitis. Each disease raises different issues with respect to efficient use of this national serological resource, but have in common the imperative to minimize testing and other costs, whilst rigorously addressing relevant surveillance objectives.

In this study, we evaluate a sampling methodology to maximize the efficient use of national slaughterhouse serological surveillance. This work is conducted in the context of archived sera from the cull cow programme to substantiate freedom from BTV infection in Ireland. The study demonstrates the use of abattoir-based serological samples collected for one large scale surveillance programme in surveillance for other bovine infections.

## Methods

### The serological samples

During the midge biting season, with the exception of very young calves, all cattle in Ireland would be expected to be outdoors on pastures, and cull cows would therefore be expected to be exposed to BTV infected to the same degree as the rest of the bovine population. Serological samples were taken from all cull cows slaughtered between 17th October and 23rd December 2016. Individual identity numbers (“tag numbers”) associated with each blood sample were recorded on Husky hand-held devices (Itronix, Portland, USA) at the slaughterhouse. Blood samples were stored at −20°C in 610 blocks, typically containing 88 samples each, filled simultaneously. All samples were stored at the Irish Government's Deparment of Food, Agriculture and the Marine (DAFM) Cork Veterinary Laboratories. Information on the block location and tag number associated with each blood sample were stored in Microsoft Excel spreadsheets.

### Sample size calculations

The sample size required to substantiate freedom from BTV was determined to be 473, given the Irish bovine population of 6.5 million (23), using a design prevalence of 2%, a sensitivity of 68.5% and specificity of 99.7%, based on a two stage testing system, which was used to optimize specificity (see section Sample Testing).

### Sample selection

Three factors guided sample selection:

- *Geographical Stratification*. Data on the herd and location of all Irish cattle on 31st December 2016 was obtained from the DAFM Animal Identification and Movement (AIM) database (23). These data were mapped in the Irish Grid Reference System using the GIS software package ArcGIS 10.3 (24), and a configuration of 45 × 45 1 km grid squares was mapped out so as to cover the locations of all herds whilst minimizing the degree to which outlying squares would contain only a few herds. The number of samples required from each grid square was calculated with reference to the proportion to the bovine population found in herds located within it, and was then rounded up to the nearest whole integer.
- *Period of Animal Residency*. Data on all cattle movements during 2016, as well as the herd and location of all Irish cattle on 31st December 2015, were obtained from the AIM database. The herd location of each animal, along with the dates of any moves from one location to another, was calculated for the period 1st April 2016 to the date of slaughter, using Transact-SQL scripts. These data were linked to the serological samples, on the basis of animal identification number. By restricting the samples tested as much as possible to animals which had been in the same location throughout the 2016 midge biting season, the geographic stratification would be truly representative of where each animal might have been exposed to BTV infection. To examine the effect this would have on the population of animals available to sample from, we used SQL scripts to calculate, from the movement data, whether each animal was in the same herd between 1st April and these times, chosen to represent a range of options: 2 months, 1 month, 2 weeks, 1 week, and 1 day prior to slaughter. We chose 1st April as the start date as it is unlikely that midges would be active before this date in Ireland (25, 26). The different periods chosen, although somewhat arbitrary, represent a trade off between the criterion which would give the most confidence that any BTV infection had been acquired in its herd of residence (1 day) and the criterion under which the largest number of animals would qualify for inclusion, whilst also giving a reasonably large probability that any BTV infection had been acquired in the herd of residence (2 months).
- *Minimizing Sample Damage*. We wanted to minimize the number of sample blocks used, as a whole block would need to be thawed if any samples were selected from it. Each thaw cycle could potentially degrade the quality of all samples in the block (27). However, the degree to which minimizing the number of blocks we used might produce a spatially clustered sample, with samples in a given block tending to come from the same herd, and also possibly aggregated around a single slaughterhouse, was unclear. To investigate this, we used Microsoft Excel to calculate how many samples came from each herd in each block, and how many samples came from each herd across the entire sample. We also used ArcGIS to map the herd location of the samples from each block. In these analyses we chose the herd that the animal was located in 1 month prior to slaughter as the reference herd for the animal. A Microsoft Excel Visual Basic for Applications (VBA) macro was written to randomize the ordering of all blocks and to sample from each in sequence until a large enough sample had been obtained from each grid square. This was iterated 1,000 times and the combination which produced the lowest number of blocks for the required sample size was selected.

### Sample testing

These samples were then tested using the IDVET Competitive ELISA Kit (Expiry 01/2018; Lot number 947), according to the manufacturer's instructions, using 40% as the cut-off point for a positive or negative result. This kit has an estimated sensitivity of 87.8% and a specificity of 98.2% (28). Any samples found to be positive were to be re-tested using the same test kit. Any samples which were found to be positive on the re-test using the same test kit were to be re-tested again using the VMRD test kit, according to the manufacturer's instructions. This test kit had a sensitivity of 70.8% and a specificity of 87.2% (29). Assuming independence between test results, the combined sensitivity and specificity of the two stage screening tests was calculated to be 68.5 and 99.7% respectively [see Dohoo et al. (30) for such calculations]. In the event of a double positive result, the sample would have been sent to the CVRL for further testing to include seroneuralization. However, this testing would have formed part of the regulatory response to a BT suspect being disclosed, and was outside the scope of the current study.

## Results

**Figure 1:**
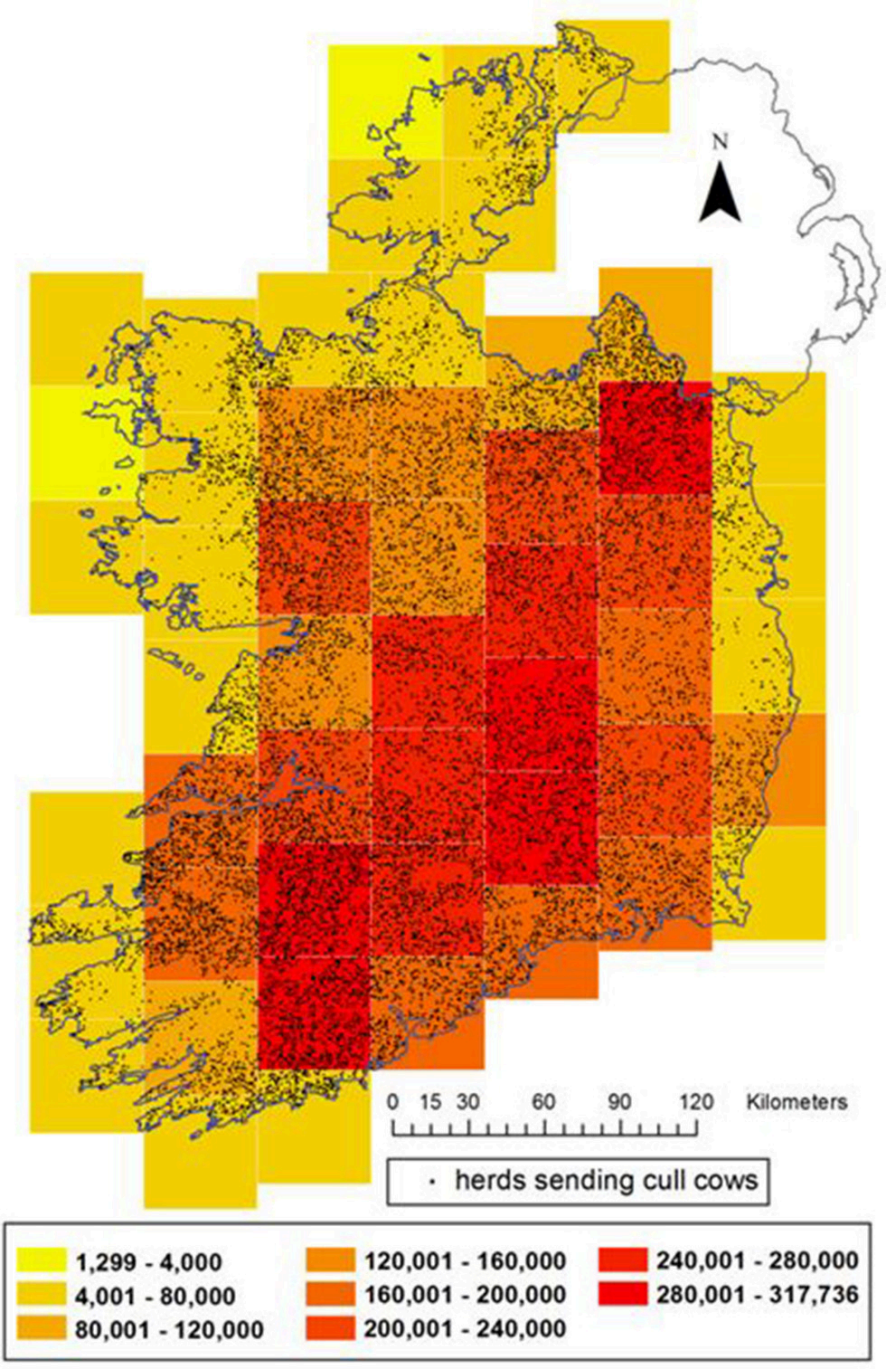
Distribution of herds sending cull cows (black dots) and the end of 2016 bovine population distribution in Ireland (color gradient on 45 km grid). For the purposes of this map, in order to conceal the location of individual farms, cull cow herds have been randomly assigned a location on land within 5 km of their real location.

In total, sera from 50,640 cull cows were available for selection for this study. The herd locations of these animals were mapped within 51 grid squares, each 45 × 45 km (Figure 1). A minimum of 14 and maximum of 3,245 came from herds located in a given square. For all maps from this study, herd locations have been randomly placed (“jittered”) on farmland somewhere within 5 km of the location of their largest fragment of land, in order to maintain farm anonymity.

**Figure 2:**
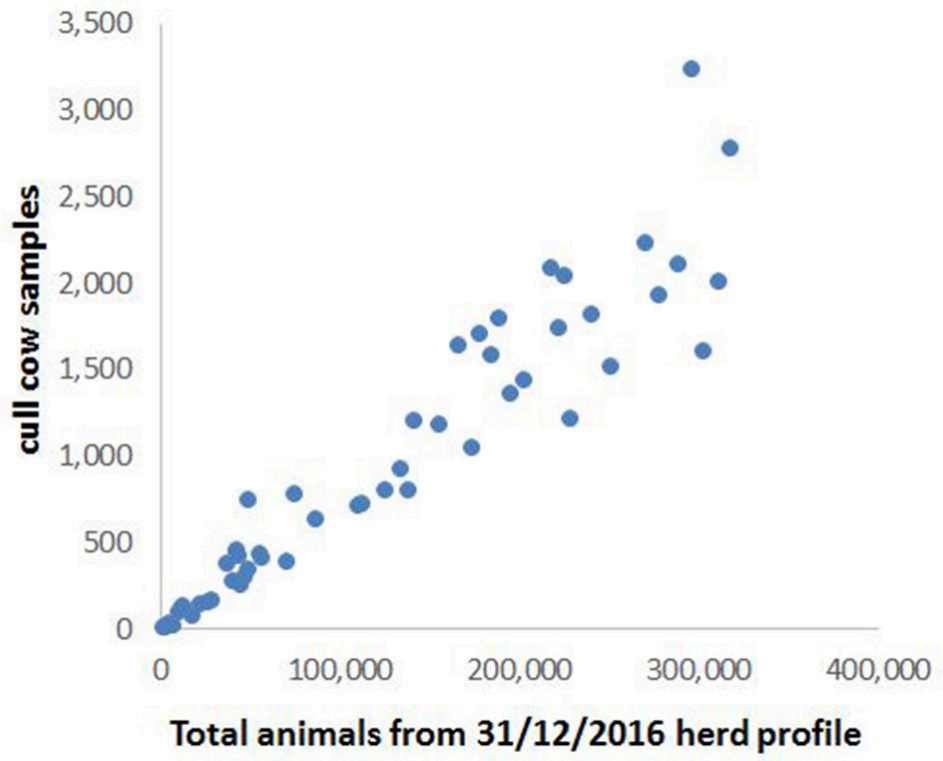
Bovine population plotted against number of cull cow samples for each of the grid squares shown in Figure 1.

There was an approximately linear relationship between the number of cull cows and the total bovine population in these grid squares (Figure 2), indicating that the spatial distribution of cull cow samples was broadly representative of the overall cattle population. Stratification according to the bovine population of each grid square, and rounding up to the nearest whole integer, gave a total of 503 samples required for testing across the 51 squares.

We found that 40,597 cows (80.2%) had remained in the same herd between 1st April and 2 months before their slaughter date, and of these only 37,519 (74.1%) remained in that herd for an additional month. This was further reduced to 35,523 (70.1%) for 2 weeks prior to slaughter, 33,624 (66.4%) for 1 week prior to slaughter, and 28,579 (56.4%) cows that remained in the same herd up to 1 day prior to slaughter. Equivalent figures for cull cows staying within the same 45 km grid cells during these periods (even though they may have moved herds) were, respectively, 43,648 (86.2%), 41,311 (81.6%), 39,844 (78.7%), 38,406 (75.8%), 34,463(68.1%).

**Figure 3:**
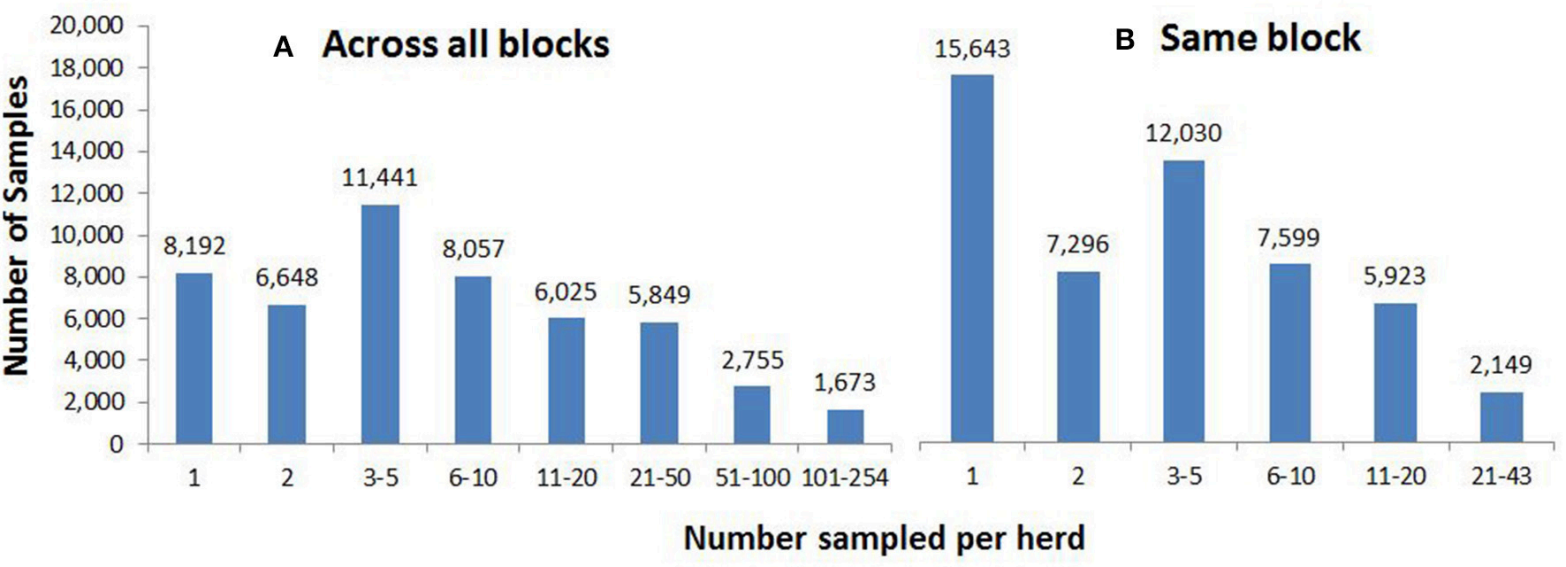
The number of samples from **(A)** the same herd, across all 610 blocks and **(B)** the same herd on the same block, according to herd location 1 month prior to slaughter.

These cull cows came from 16,394 herds, with respect to their herd location 1 month prior to slaughter. Approximately half of herds (8,192, 50.0%) were represented only once (i.e., by a single cull cow) throughout the 610 blocks (Figure 3A), whereas 10 herds had collectively culled 1,673 cows (more than 100 animals each), possibly due to movement through dealer herds (Figure 3A). Overall, almost a third (15,643, 30.9%) of samples on a given block did not share that block with any other samples from the same herd (Figure 3B), 7,296 (14.4%) samples shared their block with one other sample from the same herd, and 12,030 (23.8%) samples were on blocks containing between three and five samples from the same herd. The maximum number from the same herd on a single block was 43 (Figure 3B).

**Figure 4:**
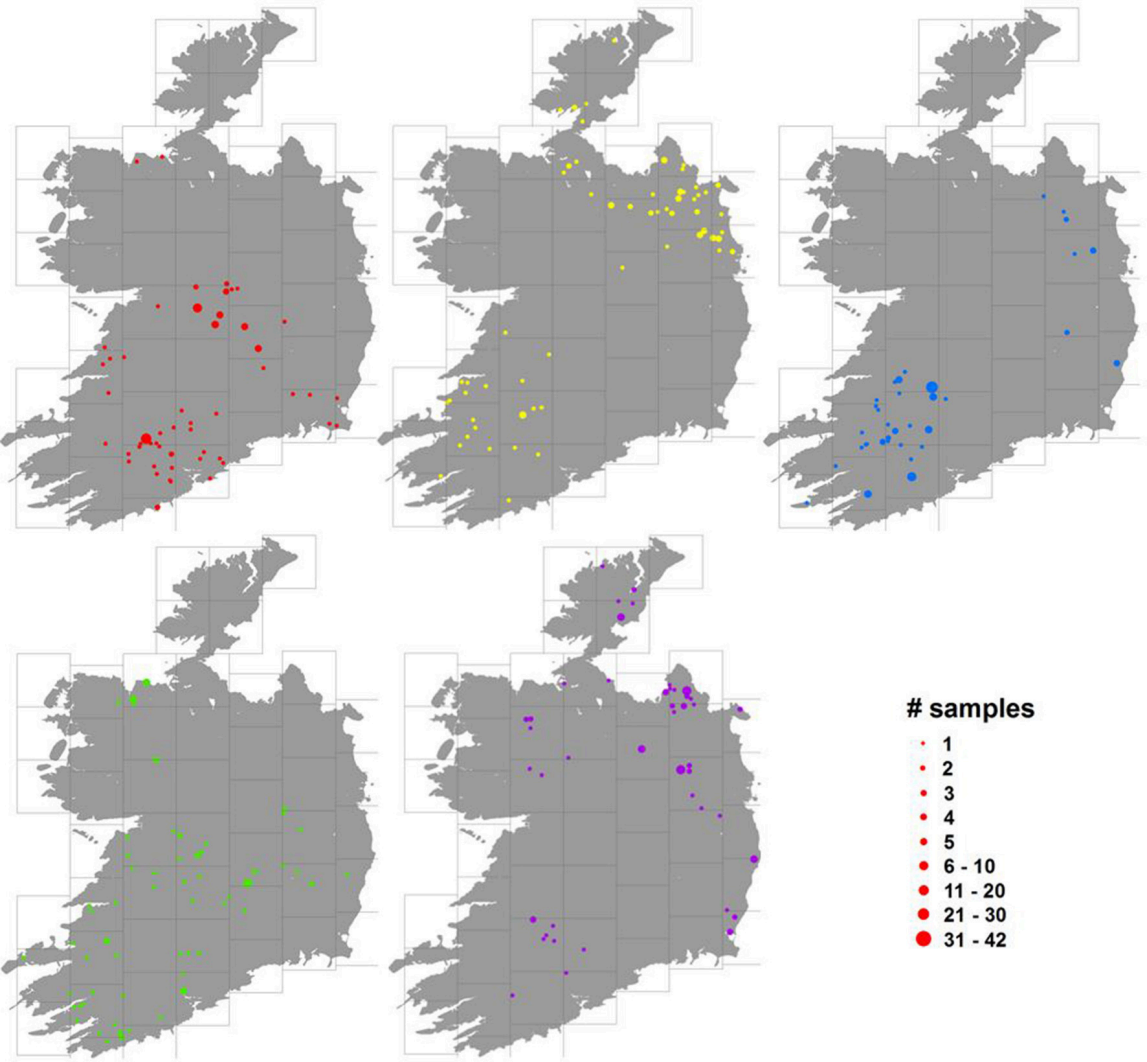
Distribution of herds of origin (herd where the animal was located 1 month prior to slaughter) for 5 randomly chosen blocks (illustrated in 5 different colors in the maps), where the size of the dots represents the number of animals sampled (see legend bottom right, which uses the red color scheme of the block illustrated in the top left figure). For the purposes of this map, in order to conceal the location of individual farms, cull cow herds have been randomly assigned a location on land within 5 km of their real location; however, this had no noticeable effect over how spatially aggregated the locations appear.

An examination of the spatial distribution of herds represented in each of the sample blocks showed that the samples tended to be clustered at a large scale, with large areas of the country not showing any herds contributing to the block. However, at smaller scales, e.g., across each 45 km grid square used to stratify the sample, there was relatively little evidence of spatial clumping. Figure 4 shows the geographical distributions across Ireland of 5 randomly selected blocks and an animation displaying similar information for all 610 blocks in sequence can be found at: *https://youtu.be/dCjDwny_w00* (farm locations in this video have been jittered in the same way as for Figures 1, 4).

On the basis of these investigations, it was decided to limit the number of animals per herd in each block to 1 animal and to include only those animals which had been in the same herd between 1st April and 1 day prior to slaughter.

**Figure 5:**
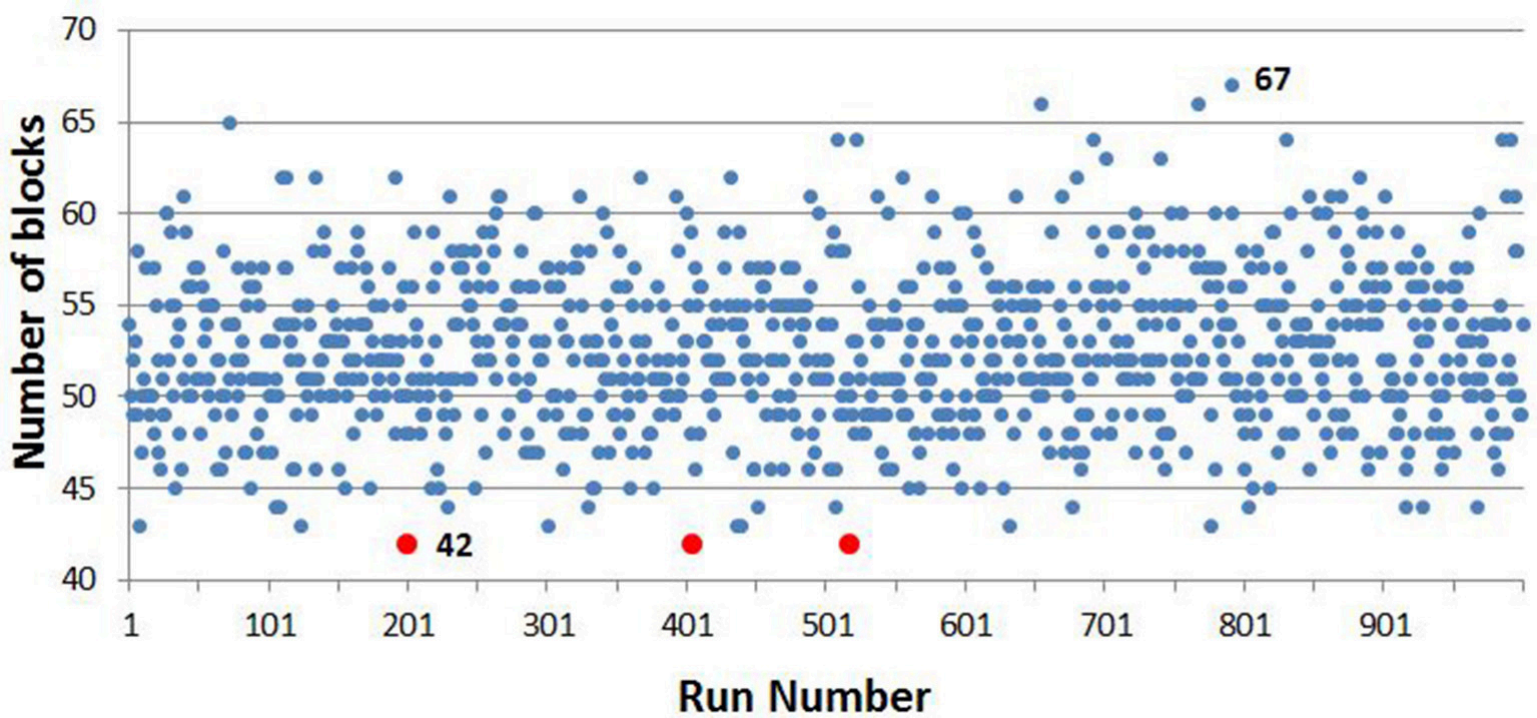
The number of blocks required, by iteration number, over 1,000 iterations, with the minimum and maximum number indicated. The minimum number, 42, was met on 3 of the iterations, which are highlighted in red.

Over the 1,000 runs of the sample selector VBA script, the minimum number of blocks from which the sample could be obtained was 42, which was selected three times, the first time at the 200th run, which was the one used to provide a list of samples for serological testing for BTV. The maximum number of blocks selected in any iteration of the VBA script was 67. The distribution of the number of blocks selected at each iteration of the script is shown in Figure 5.

Serological analysis of the selected samples found that all 503 samples tested were negative for BTV antibody, thereby providing assurance of freedom of exposure to BTV in the Irish cattle population in the 2016 vector season. All samples were negative on the initial test and there was therefore no requirement to conduct further testing.

## Discussion

This study found no evidence of seroconversion to BTV in the 2016 vector competent season in a geographically stratified sample of sera collected from cull cows at slaughter. This provides assurance for freedom from BTV infection in cattle in Ireland during this period.

In this study, archived cull cow sera were used as the source of serological samples. A sufficiently large number of samples were needed to ensure that stratified selection was possible from each 45 km grid square. A key component of this was recording of individual identity numbers for each animal at slaughter and the ability to link these numbers both to the stored serological samples and to the births and movement data. As these data will be available in every year at low cost, this study provides a basis for their use on an ongoing basis, to establish the presence or absence of BTV in Ireland as well as for other viral diseases which might not otherwise be detected, such as enzootic bovine leukosis (EBL) (31).

We were able to obtain the 503 samples needed using only 42 out of a possible 610 sample blocks, whereas the majority of blocks would have had to be thawed if the sample was drawn in a completely random manner. It was important to minimize the number of blocks both because of the detrimental effect of repeated thawing and freezing on all thawed samples and to reduce the demands on labor within the laboratory. We therefore used a technique which took the maximum number of samples from each block used, with the conditions that the sample should be stratified with reference to the spatial distribution of the bovine population in Ireland and that not more than one sample should come from the same herd in the same block. The sampling was made more efficient by choosing the minimum number of blocks needed from a run of 1,000 iterations. The fact that this number, 42, was arrived at on three occasions suggests that the process would not be greatly improved by increasing the number of iterations. We note that these concerns would not have arisen if serological samples had been stored in individual vials rather than blocks.

A two stage test screening procedure was envisaged, with samples positive to the IDVET Competitive ELISA being retested using the VMRD test. This strategy was to be used to minimize the number of false positives, and thereby optimize the specificity of the study. In practice, however, all samples were negative on the initial screening test, and no retesting was conducted. With the use of a single screening test, test sensitivity is maximized and concerns are avoided about the potential for dependence between the IDVET Competitive ELISA and the VMRD test, which has implications when estimating the performance of tests when conducted in series.

The legislative requirement upon which the sampling plan was based required the collection of samples from each 45 × 45 km grid square in Ireland. The storage of serological samples from a large number of slaughterhouses, referenced to the tag number of the animal, has allowed access to data from a large geographical spread of locations needed for this type of study. We restricted our samples to animals which had remained in the same herd between 1st April and the day prior to slaughter in 2016, in order to ensure that any BTV infection would have been acquired in the grid cell assigned to the animal. However, the condition could have been relaxed to include animals that changed herd whilst remaining in the same grid cell throughout the period, which in this study would have allowed us to select our samples from a larger population of cull cows. Similarly, in situations where animals are moved to assembly herds several days before slaughter, the criterion might be relaxed to include animals moved up to 1 week prior to slaughter, as any immune response to BTV (necessary for a positive test) would take time to become evident—Eschbaumer et al. report seroconversion in cattle approximately 1 week after experimental infection with BTV-8 (32).

Our sample size calculation is based on an assumption of close to uniform prevalence, whereas it might be argued that a vector-borne infection such as bluetongue might initially become established on a more localized or regional basis, similar to the distribution of infection due to Schmallenberg virus in Ireland in 2012 (33). For such a situation, the number of samples might be insufficient to establish freedom from infection, especially when one considers that the number of samples per grid square is proportionate to the number of cattle located in herds within the square: in the case of some of the squares as few as 1 animal was sampled (although it should be noted that these sparsely populated grid squares tended to be in the west of the country, where the risk of the incursion of a wind borne disease is thought to be considerably less). In contrast, a risk-based approach to sampling may be warranted, where samples from the areas at greatest risk of airborne incursion of bluetongue virus should be subjected to more intensive sampling, as directed by meteorological modeling (34). With such an approach, the potential for introduction of infection through the importation of infected animals will also need to be considered.

The legislation underpinning bluetongue surveillance requires sampling after the end of the vector season. Our study was based on samples collected from animals slaughtered from 17th October until 23rd December. While vector activity was possible at the earlier stages of this sampling period, it was considered to be minimal at that stage of the year, and therefore unlikely to interfere with the serological status of the animals sampled. Both McCarthy et al. (25) (working in Ireland), and Jess et al. (in Northern Ireland) found the midge vector season occurred from April to December, but there was a considerable fall off in vector activity from the end of October onwards (25, 26). The pattern of cull cow slaughter in Ireland is largely seasonal, with large numbers slaughtered off pasture at the end of the grazing season. Therefore, these animals would have been exposed to the midge population in the months prior to slaughter. There are no wild regions in Ireland without the presence of cattle, so it is unlikely that BTV could avoid detection in a wildlife host.

## Implications

This study has shown that Ireland was free of BTV in 2016. The findings can be also be used to provide assurance of freedom from BTV infection in sheep, as midges have been found to be more attracted to cattle than sheep (35, 36). Furthermore, a previous study confirmed higher levels of seroconversion to Schmallenberg virus, another midge-borne viral infection, among cattle (37).

This study shows the suitability of the Irish cull cow serological sample archive as a basis for establishing country-wide freedom from infection, and, more generally, provides a roadmap for how such surveillance resources could be used in Ireland to provide assurance of freedom and to calculate prevalences for a range of endemic diseases. It has also been shown how samples collected for one disease surveillance programme can be stratified geographically to give representation for providing assurances of freedom for other infectious agents. This could make the execution of such programmes more efficient and cost effective.

## Author contributions

JT devised the methodology, conducted the analyses and had overall responsibility for writing the manuscript. DB and SM both played major roles in initiating the study, maintained oversight of it from inception to conclusion, helped to devise the methodology, provided expertise and advice throughout the study and substantially contributed to the writing of the manuscript. TC devised the sampling methodology and calculated the required sample size and GM created the jittered geo-referencing of the locations of dairy herds used for some of the figures. TC, RO, GM, and EL contributed their expertise to the study and contributed to the writing of the manuscript.

### Conflict of interest statement

The authors declare that the research was conducted in the absence of any commercial or financial relationships that could be construed as a potential conflict of interest.
